# RETRACTION: Augmented venoarteriolar response with ageing is associated with morning blood pressure surge

**DOI:** 10.1113/eph.13639

**Published:** 2024-09-20

**Retraction**: J.‐K. Yoo, D.‐D. Sun, R. S. Parker, M. A. Urey, S. A. Romero, J. S. Lawley, S. Sarma, W. Vongpatanasin, C. G. Crandall, and Q. Fu. (2018). Augmented Venoarteriolar Response with Ageing is Associated with Morning Blood Pressure Surge. *Experimental Physiology 103*(11), 1448‐1455. https://doi.org/10.1113/EP087166

The above article, published online on 20 August 2018 in Wiley Online Library (wileyonlinelibrary.com), has been retracted by agreement between the authors; the journal Editor in Chief, Damian Bailey; The Physiological Society; and John Wiley and Sons Ltd. The authors reported that hypertensive patients had been included by mistake in the database from which the data for this study were drawn. The retraction has been agreed to because the editors and authors agree that these errors substantially altered the results and conclusions of this article.

